# The science of ultrasound therapy for fracture healing

**DOI:** 10.4103/0019-5413.50845

**Published:** 2009

**Authors:** Gregory J Della Rocca

**Affiliations:** Department of Orthopaedic Surgery, University of Missouri, Columbia, MO 65212, USA

**Keywords:** Ultrasound, fracture healing, nonunion

## Abstract

Fracture healing involves a complex interplay of cellular processes, culminating in bridging of a fracture gap with bone. Fracture healing can be compromised by numerous exogenous and endogenous patient factors, and intense research is currently going on to identify modalities that can increase the likelihood of successful healing. Low-intensity pulsed ultrasound (LIPUS) has been proposed as a modality that may have a benefit for increasing reliable fracture healing as well as perhaps increasing the rate of fracture healing. We conducted a review to establish basic scince evidence of therapeutic role of lipus in fracture healing. An electronic search without language restrictions was accomplished of three databases (PubMed, Embase, Cinahl) for ultrasound-related research in osteocyte and chondrocyte cell culture and in animal fracture models, published from inception of the databases through December, 2008. Studies deemed to be most relevant were included in this review. Multiple in vitro and animal in vivo studies were identified. An extensive body of literature exists which delineates the mechanism of action for ultrasound on cellular and tissue signaling systems that may be related to fracture healing. Research on LIPUS in animal fracture models has demonstrated promising results for acceleration of fracture healing and for promotion of fracture healing in compromised tissue beds. A large body of cellular and animal research exists which reveals that LIPUS may be beneficial for accelerating normal fracture healing or for promoting fracture healing in compromised tissue beds. Further investigation of the effects of LIPUS in human fracture healing is warranted for this promising new therapy.

## INTRODUCTION

Despite advances in fracture fixation and treatment, difficulties with fracture healing persist. Fracture nonunion is a source of disability and carries with it significant socioeconomic costs.^1^ As the world population increases and as technological advances enable higher velocity travel, incidence of fractures can be expected to increase worldwide. With this increase, incidence of fracture nonunion is also expected to increase. Increasing both the reliability of fracture healing as well as the rate of fracture healing remain goals which have yet to be attained in practice. Furthermore, assisting fracture healing in compromised patients (those with open fractures, tobacco users, end-stage diabetics, end-stage renal disease patients) is also attractive. In the United States, fracture healing fails or is delayed 5–10% of the time.^2^–^4^

Multiple methods of accelerating fracture healing have been proposed, and some are approved for use in the clinical setting. Bone morphogenetic proteins, implanted at the site of open tibia fractures, have been shown to improve rates of fracture healing and decrease rates of infection.^5^ Electrical stimulation, either by implanted direct current stimulators or by externally applied capacitative or inductive couplers, has been variably shown to be marginally effective.^6^,^7^

Low-intensity pulsed ultrasound (LIPUS) has been proposed as a method for promoting fracture healing. Historically, ultrasound was contraindicated in the setting of fractures. Much of this arose from animal data demonstrating that ultrasound damaged bones or delayed fracture healing.^8^ Other groups, however, demonstrated contradictory results.^9^,^10^ A growing body of the literature, reviewed here, has demonstrated that ultrasound intensity appears to determine whether its effect on fracture healing is beneficial or detrimental, with LIPUS apparently demonstrating beneficial effects.

One commercial device (EXOGEN^®^, Smith and Nephew, Memphis, TN) is already approved by the United States Food and Drug Administration (FDA) for the treatment of fresh fractures (claiming to accelerate fracture healing rates by up to 38%) and also for use in fractures at risk for nonunion. Development of this device was tied to a body of basic science evidence that points toward its potential therapeutic role in fracture healing. This manuscript intends to review this evidence. A recent meta-analysis suggests that use of LIPUS significantly reduced healing time for nonoperatively managed fractures in humans.^2^ Further prospective data will be needed to further evaluate its efficacy in accelerating fracture healing and in decreasing nonunion rates in the clinical setting.

## CELLULAR EFFECTS OF ULTRASOUND ON OSTEOCYTE AND CHONDROCYTE LINEAGES

### Ultrasound stimulation of calcium turnover

Early work by Ryaby *et al*. on the effects of LIPUS on osteoblasts demonstrated increased calcium incorporation in cultured bone cells after treatment with ultrasound.^11^ Also, Ryaby's group presented that transforming growth factor-β (TGF-β) synthesis and adenylyl cyclase activation in osteoblasts are also stimulated by LIPUS.^12^ How does this happen?

Bone cells can both perceive and respond to mechanical forces via signal transduction mechanisms. Research on this signal transduction mechanism and its relationship with responsiveness to LIPUS was further stimulated by a finding of ultrasound effect on bone formation by Nolte *et al*. in an *ex vivo* metatarsal rudiment model.^13^ Cartilaginous metatarsal rudiments from 17-day-old fetal mice were resected en bloc and cultured for 1 week with or without stimulation with LIPUS. Growth of the calcified portion of the metatarsal diaphysis in LIPUS-treated metatarsal rudiments was noted to triple during the week of treatment as compared to the control, untreated group (530 μm versus 180 μm). Nolte's group concluded that LIPUS directly affects osteoblasts and ossifying cartilage, resulting in more active ossification.

Direct examinations of LIPUS effects on an osteosarcoma cell line were accomplished by an Australian group in 2001.^14^ The osteosarcoma cell line, UMR-106, was subjected to 20-min doses of LIPUS. Expression of the immediate early-response genes c-fos and cyclooxygenase-2 (COX-2) were up-regulated by this treatment. Elevated mRNA levels for alkaline phosphatase and osteocalcin were also noted after LIPUS treatment. These findings are suggestive of a beneficial effect of LIPUS on fracture repair and bone formation.

### Effect of ultrasound on prostaglandin signaling pathways

Prostaglandins are abundant in bones. They are produced by osteoblasts and are up-regulated at sites of fractures.^15^ Prostaglandin E_2_ (PGE_2_) production is regulated by COX-2. Both are thought to be vital for fracture healing, and can be affected by treatment with LIPUS. The effect of LIPUS on PGE_2_ regulation and on COX-2 mRNA expression was recently examined in the mouse osteoblastic cell line, MC3T3-E1.^16^ Kokubu *et al*. demonstrated a three-fold increase in the amount of PGE_2_ produced by cells exposed to LIPUS for 60 min, as compared to control cells. Up-regulation of COX-2 was also noted after treatment of the cells with LIPUS; this up-regulation peaked at 60 min and subsided by 180 min.

PGE_2_ stimulates cellular responses by signaling through a cascade of enzymatic reactions. PGE_2_ binds to a guanine nucleotide-binding (G) protein-coupled receptor (GPCR), a well-studied class of heptahelical transmembrane receptors involved in cellular responses to external stimuli as diverse as photons, odorants, hormones (such as glucagon), and other extracellular mediators (such as epinephrine).^17^ The GPCRs, upon activation by ligand binding, activate diverse downstream pathways of intracellular signaling, ultimately leading to expression of certain genes associated with intracellular metabolism and with cell growth and proliferation.^18^

If PGE_2_ production is related to fluid shear (thought to be created by LIPUS), then up-regulation of the downstream effectors of PGE_2_ should be similarly affected. Two studies have focused on this specific relationship. Wadhwa *et al*. described the effect of fluid shear stress on COX-2 gene expression in cultured MC3T3-E1 osteoblasts as well as in primarily cultured osteoblasts from mouse calvaria.^19^ They demonstrated that fluid shear stress increased COX-2 gene expression within 30 min. This appeared to be under the direct control of extracellular signal-regulated kinase (ERK), one of the mitogen-activated protein (MAP) kinases known to be activated by growth factor receptors and by GPCRs, such as the PGE_2_ receptor.

### Ultrasound-mediated gene regulation and chondrocyte differentiation

Further examination of the effect of LIPUS on osteogenic gene regulation was undertaken by Chen *et al*., who utilized LIPUS as the mechanism by which fluid shear/mechanical stimulation of cells was accomplished.^20^ Treatment of human ostoblasts with LIPUS resulted in an elevation of mRNA levels for Cbfa1/Runx2 and for osteocalcin, necessary for osteogenesis. LIPUS also increased activation of ERK via a cascade resulting in its phosphorylation. Messenger RNA expression for Cbfa1/Runx2 and osteocalcin induced by LIPUS was successfully blocked by pretreatment of cells with pertussis toxin, a specific inhibitor of the inhibitory G protein, Gi. Pertussis toxin also successfully reduced LIPUS-mediated ERK phosphorylation. Treatment with a specific inhibitor of ERK also abrogated Cbfa1/Runx2 and osteocalcin mRNA up-regulation by LIPUS stimulation. Chen *et al*. concluded that both membrane-coupled G proteins and intracellular MAP kinases are important LIPUS-mediated up-regulation of genes involved in osteogenesis in human osteoblasts.

Secondary fracture healing occurs through formation of a cartilaginous callus, which is then converted to osseous callus. At fracture sites, mesenchymal stem cells are differentiated into chondrocytes during the formation of the soft, cartilaginous callus. Mesenchymal stem cells, in primary culture, can be differentiated into chondrocytes by treatment with TGF-β. Aggrecan deposition, indicative of chondrocyte differentiation, was increased (8.7-fold versus 2.3 fold) if LIPUS was added to TGF-β, without increasing total protein content or cellular proliferation. This is consistent with the hypothesis that ultrasound increases chondrocyte differentiation of mesenchymal stem cells treated with TGF-β.^21^

Chondrocytes can be induced to proliferate and maintain chondrocytic lineage also via treatment with LIPUS. Chondrocytes from neonatal Wistar rats were isolated and cultured to examine this phenomenon.^22^ Exposure to LIPUS induced and maintained expression of type II collagen and aggrecan, and blocked expression of type X collagen. It was noted to increase expression of TGF-β. Addition of TGF-β to the nontreated cells stimulated similar results, and blockade of TGF-β by addition of a neutralizing antibody abrogated the results seen after LIPUS stimulation. The authors concluded that the mechanism of action of LIPUS likely involves TGF-β, in addition to other mechanisms, and that TGF-β is vital for chondrocyte responsiveness to LIPUS.

### Ultrasound-mediated gene regulation and osteocyte differentiation/osteogenesis

Leung *et al*. recently studied the effects of LIPUS on cultured human periosteal cells.^23^ After obtaining normal human periosteum, and creating a primary cell culture, the cells were treated with 5, 10, or 20 min of LIPUS daily for 2 or 4 days each. Untreated cells served as the controls. After 2 days of LIPUS treatment, cell proliferation was enhanced as compared to untreated controls. Osteocalcin expression was increased after 4 days of treatment. Alkaline phosphatase production and vascular endothelial growth factor expression were both increased after either 2- or 4-day LIPUS treatment regimens. Heightened expression of these factors trended toward maximal at 20 min of daily LIPUS treatment. After 4 weeks of treatment, calcium nodule formation was dramatically increased in the LIPUS-treated cultures as compared to controls. The authors concluded that treatment of periosteum with LIPUS resulted in osteoblastic differentiation, and they opined that the treatment should be started clinically as soon as possible following fracture.

It is thought that the response of bone cells to mechanical stimulation is dependent upon both the frequency and the amplitude of stimulation.^24^,^25^ Naruse *et al*., examining low-strain, high-frequency mechanical stimulation in osteoblasts by using LIPUS, demonstrated results consistent with bone formation.^26^ Primary cultures of rat osteoblasts and osteocytes were exposed to LIPUS for 20 min. Up-regulation of osteocalcin and COX-2 expression were noted in response. Blockade of a MAP kinase pathway (p38) associated with osteogenic differentiation abrogated this up-regulation, but blockade of a MAP kinase pathway (ERK) associated with cell proliferation did not. Blockade of phosphatidylinositol 3-kinase (PI3K) pathways, which are upstream regulators of p38 MAP kinases, also abrogated osteocalcin and COX-2 up-regulation. It is thought that PI3K is vital for bone formation through nitric oxide (NO) production, and it is also under the control of integrins, present within focal adhesion complexes. Although it is difficult to reconcile these results with aforementioned findings by Chen *et al*.,^20^ it is felt that this explains the potential efficacy of LIPUS for fracture healing, that is, osteogenic differentiation, but not osteocyte proliferation, is necessary.

A landmark publication by Tang *et al*. nicely synthesized the complex interplay of intracellular signaling mechanisms and how they function for cellular responsiveness to LIPUS in both the MC3T3-E1 osteoblastic cell line and in primary osteoblasts cell culture from fetal rat calvaria.^27^ LIPUS directly stimulated increases in integrin subunit expression on cell surfaces, COX-2 expression, and PGE_2_ expression. Anti-integrin antibodies attenuated this osteoblast responsiveness to LIPUS, implying the importance of integrin signaling for LIPUS stimulation. Inhibitors of PI3K had similar effects as anti-integrin antibodies, indicating that PI3K is an important downstream signaling effector. Phosphorylation of focal adhesion kinase (FAK), which is associated with its activation and shown to be affected by GPCR stimulation,^28^ as well as phosphorylation of ERK, PI3K, and Akt were also promoted by exposure to LIPUS. Dominant-negative forms of FAK, ERK, PI3K (p85 subunit), and Akt, when transfected into the osteoblasts lines, abrogated activation of the COX-2 promoter after stimulation with LIPUS, and growth of mineralized nodules in these transfected cells was significantly inhibited. In conclusion, stimulation of the COX-2 promoter by LIPUS was felt to be under the control of integrins, FAK, PI3K, ERK, and Akt, representing the most comprehensive description of signaling systems involved in osteoblasts' LIPUS sensitivity to date.

Vascular endothelial growth factor (VEGF) is also important for skeletal development,^29^ and exogenous VEGF can enhance bone formation in murine femur fractures when administered at the site of fracture.^30^ Angiogenesis is an important component for fracture healing as well. VEGF production related to LIPUS was examined recently in human osteblastic cell lines by Wang *et al*.^31^ Cells were subjected to 20 min of LIPUS. Production of VEGF messenger RNA and protein, NO, and hypoxia-inducible factor-1α (HIF-1α) were all stimulated by LIPUS. Inhibition of NO production reduced the VEGF response to LIPUS. Wang's group concluded that the VEGF and HIF-1α responses to LIPUS are mediated by nitric oxide (NO) production. Further research on this responsiveness demonstrated that integrin receptors, present in focal adhesions, are vital for this signaling cascade;^32^ if LIPUS stimulates osteogenic responsiveness via mechanical stimulation, focal adhesions (mediating cellular contact with the surrounding media) are a reasonable structure to implicate in this responsiveness.

Collagen is a necessary substrate for normal bone formation. Saito *et al*. recently undertook an examination of two different ultrasound intensities (low, 30 mW/cm^2^, and high, 120 mW/cm^2^) in cultured MC3T3-E1 osteoblasts.^33^ They demonstrated an accelerated formation of collagen fibers with a packing conducive to bone formation by osteoblasts treated under the low intensity arm. The higher intensity treatment did not lead to this type of collagen fiber formation. Similarly up-regulated by the low-intensity ultrasound were COX-2, lysyl oxidase, telopeptidyl lysyl hydroxylase, and helical lysyl hydroxylase, the latter three enzymes being important for proper collagen cross-linking.

## ANIMAL STUDIES OF ULTRASOUND-ASSISTED FRACTURE HEALING

Although *in vitro* research on the mechanisms of fracture healing and osteocyte/chondrocyte stimulation by LIPUS is compelling, live animal and human studies are necessary to determine the utility of LIPUS in an *in vivo* setting. It is entirely possible that laboratory research *in vitro* will not be supported by *in vivo* data. In this manuscript, we will focus specifically on nonhuman *in vivo* animal studies. A separate manuscript in this issue will deal primarily with research on LIPUS in human fracture healing.

While ultrasound has been used to quantify fracture callus formation and to assist with diagnosis of nonunions, its use to stimulate fracture healing in animal models has only recently been explored. One of the earliest English language publications related to the use of ultrasound to assist fracture healing in an animal model was published in 1983.^34^ Bilateral transverse fibular fractures were created in adult Wistar rats. Ultrasound was administered to one of the bilateral fractures in each rat, at an intensity of 500 W/cm^2^ for 5 min at a time, 4 days a week, at different stages post-fracture. They demonstrated the greatest effectiveness of ultrasound treatment to be present when ultrasound was delivered during the first 2 weeks postfracture.

Another study on fibular osteotomy healing, in a New Zealand white rabbit model, and its influence by LIPUS was published in 1990.^35^ One hundred thirty-nine rabbits underwent bilateral fibular osteotomy, and one side in each rabbit underwent treatment with 20 min of LIPUS daily (the contralateral fibula served as a control). By post-osteotomy day 17, LIPUS-treated fibular osteotomies had reached strengths equal to intact fibulae, while untreated control osteotomies did not reach such strength until post-osteotomy day 28. Use of LIPUS in human patients with fractures was proposed as a possible modality to decrease fracture healing times.

Gene expression *in vivo* has also been studied in animal fracture models treated with LIPUS. Yang *et al*. created bilateral closed femur fractures in 79 skeletally mature Long-Evans rats and treated one side in each rat with LIPUS for 20 min per day.^36^ At 3 weeks, average torsional stiffness and maximum torque were significantly greater in LIPUS-treated fractures than in the contralateral control fractures. Assays of fracture calluses failed to show differences in calcification, cell number, or collagen content between LIPUS-treated and control fractures. However, aggrecan expression in LIPUS-treated fractures was (significantly) higher on postfracture day 7 than in controls, as was expression of aI(II) procollagen (the latter trended higher but did not reach statistical significance). The authors suggested that the increased mechanical properties in LIPUS-treated fractures may be related to earlier stimulation of extracellular matrix protein formation.

If LIPUS is helpful for fracture healing in animal models, at which stage of healing does it demonstrate beneficial effects? An investigation of this phenomenon in skeletally mature Long-Evans rats was accomplished by Azuma *et al*.^37^ Bilateral closed femur fractures were created in 69 rats, and one side for each rat was treated with LIPUS for 20 min per day according to the following regimen: one group received treatment on days 1–8, the second group on days 9–16, the third group on days 17–24, and the fourth group on days 1–24 (throughout the experimental period). All animals were sacrificed on day 25. Maximum torque of LIPUS-treated fractures was greater than untreated controls in all groups, despite no variance in hard callus area or bone mineral content at the fracture site. These findings suggested that LIPUS may have a beneficial effect during all phases of fracture healing.

LIPUS has been demonstrated to have a beneficial effect on fracture healing in many further studies involving multiple animal models. Use of LIPUS for 20 min per day for 20 days was noted to improve mechanical properties, histology, and radiographic findings in New Zealand white rabbits, as compared to control non-LIPUS-treated animals, after creation of a unilateral mandibular osteotomy.^38^ Healing of a midshaft tibia fracture model in sheep was noted to be more rapid (79 versus 103 days) after application of LIPUS for 20 min daily, and mean cortical bone mineral density and ultimate bending strength at 75 days were noted to be greater in the LIPUS-treated tibiae (although no difference existed at 120 days).^39^ LIPUS was also examined in a diabetic Wistar rat fracture model, where LIPUS-treated femoral fractures in diabetic rats demonstrated greater torsional stiffness and torque to failure at 6 weeks, relative to non-LIPUS-treated controls.^40^

## CONCLUSIONS

Great interest has arisen in the utility of LIPUS for the treatment of fresh fractures and fracture nonunions in patients. A great deal of research on molecular mechanisms of action for LIPUS that may lead to improved fracture healing has been accomplished. Although research on these mechanisms of action is in its infancy, a great deal has been elucidated. We have a greater understanding of the complex interplay of mechanical stimuli (LIPUS), cellular attachments to surrounding matrices (via focal adhesions and integrins), intracellular signaling cascades, and gene expression for chondrocyte- and osteocyte-specific proteins. Much research remains to be done in cell culture, to delineate these specific pathways even further, in animal models, to reinforce what *in vitro* results have demonstrated, and (ultimately) in patients, primarily to determine whether LIPUS as a modality to treat fractures is worthwhile and cost effective.
